# Temporal Properties of Self-Prioritization

**DOI:** 10.3390/e26030242

**Published:** 2024-03-09

**Authors:** Zhuoen Lu, Xun He, Dewei Yi, Jie Sui

**Affiliations:** 1 School of Psychology, University of Aberdeen, Aberdeen AB24 3FX, UK; jie.sui@abdn.ac.uk; 2 Department of Psychology, Faculty of Science and Technology, Bournemouth University, Poole BH12 5BB, UK; xhe@bournemouth.ac.uk; 3 School of Natural and Computing Sciences, University of Aberdeen, Aberdeen AB24 3FX, UK; dewei.yi@abdn.ac.uk

**Keywords:** self, perceptual matching, dynamic connectivity, EEG, social context

## Abstract

Using electroencephalogram (EEG), we tested the hypothesis that the association of a neutral stimulus with the self would elicit ultra-fast neural responses from early top-down feedback modulation to late feedforward periods for cognitive processing, resulting in self-prioritization in information processing. In two experiments, participants first learned three associations between personal labels (self, friend, stranger) and geometric shapes (Experiment 1) and three colors (Experiment 2), and then they judged whether the shape/color–label pairings matched. Stimuli in Experiment 2 were shown in a social communicative setting with two avatars facing each other, one aligned with the participant’s view (first-person perspective) and the other with a third-person perspective. The color was present on the t-shirt of one avatar. This setup allowed for an examination of how social contexts (i.e., perspective taking) affect neural connectivity mediating self-related processing. Functional connectivity analyses in the alpha band (8–12 Hz) revealed that self–other discrimination was mediated by two distinct phases of neural couplings between frontal and occipital regions, involving an early phase of top-down feedback modulation from frontal to occipital areas followed by a later phase of feedforward signaling from occipital to frontal regions. Moreover, while social communicative settings influenced the later feedforward connectivity phase, they did not alter the early feedback coupling. The results indicate that regardless of stimulus type and social context, the early phase of neural connectivity represents an enhanced state of awareness towards self-related stimuli, whereas the later phase of neural connectivity may be associated with cognitive processing of socially meaningful stimuli.

## 1. Introduction

Social environments require humans to develop self-awareness and prioritize self-related information to achieve optimal behavior. This acquired knowledge guides human behavior in a powerful way. For instance, stimuli associated with the self are perceived rapidly and automatically, facilitating an accelerated uptake of information [1,2,3,4]. This self-prioritization effect persists even when stimulus contrast is degraded, indicating that stimuli self-relatedness enhances information processing through heightened self-awareness [5]. Neuroimaging research in humans has shown that neural activity is not a completely accurate reflection of what is presented in the external environment but is a reflection of internalized representation of these external stimuli (e.g., [6]). Understanding how self-awareness mediates neural activity during information processing, specifically in the early stages of processing, may be particularly important for understanding human interaction in social environments in which they must take immediate responses to stimuli based on their personal relevance and not just their perceptual salience.

One explanation of self-prioritization effects is that the self-relatedness of stimuli enhances their social salience through the activation of self-representation, which in turn modulates attention and decision making, subsequently influencing task performance [7,8,9,10], but an explanation of when this self-saliency occurs at particular early time scales and how it is linked to specific neural mechanisms is lacking. Humphreys and Sui (2016) proposed a self-attention network (SAN) to understand the neural mechanisms of self-prioritization effects [11]. Within this SAN, self-prioritization emerges through the ventral neural network, including the ventral prefrontal cortex, VMPFC, and the posterior superior temporal sulcus, pSTS, which interacts with top-down control processing mediated through the dorsal attentional control (frontoparietal) network. The VMPFC mediates the influence of internal self-representation [12,13,14,15,16,17], while the pSTS reflects responses to socially salient external stimuli [18,19,20]. Activity in the dorsal attention network, typically required for more difficult tasks [21,22], is increased during the processing of other related information that demands increased attentional efforts, but this is not observed during self-related processing [23].

The SAN model has been supported by functional magnetic resonance imaging (fMRI) studies and neuropsychological work. For example, in a recent fMRI study, a dynamic causal modeling analysis revealed that self-related processing was associated with an enhanced excitatory connection from the VMPFC to the left pSTS [23], indicating that referring a stimulus to the self activates internal self-representation by the VMPFC, which immediately drives attention towards the stimulus through the mediation of the left pSTS. In contrast, the processing of less socially salient stimuli was associated with enhanced activity in the dorsal attentional control network. Neuropsychological evidence has demonstrated that brain damage in the VMPFC results in hypo self-prioritization [24], consistent with a loss of self-influence after brain damage in the ventral network. Conversely, brain damage to the dorsal frontoparietal network leads to hyper self-prioritization, suggesting the release of attentional control exaggerating self-prioritization [24]. This relationship between self-prioritization and control processing has also been found in healthy participants (e.g., [25]). The evidence indicates that there is a relationship between bottom-up perception and top-down processes during self-related processing. Yet, the dynamics that drive these self-prioritization effects remain unclear.

The dynamic processing of self-related stimuli may be linked to the two dimensions of consciousness, involving the temporo-spatial expansion of early-stimulus-induced activity and the temporo-spatial globalization of late-stimulus-induced activity, as proposed by Northoff and colleagues in their temporo-spatial theory of consciousness (TTC) [26,27,28]. The authors elucidate different dimensions of consciousness, highlighting how the brain’s spontaneous activity and its spatial-temporal dynamics influence responses to external stimuli. This interaction is crucial in shaping the spatial and temporal characteristics of consciousness, facilitating the integration of sensory experiences. The link between stimulus-evoked brain activity and the temporo-spatial expansion for phenomenal consciousness is characterized by distinct changes in neural responses to stimuli, compared to unconscious processing. For example, the greater the amplitude of neural activity in response to an external stimulus, the more likely it is associated with consciousness. However, these dynamic changes in neural activity are not simply additive effects of pre- and post-stimulus activity but represent a complex interaction [29]. On the other hand, there is evidence that the self-relatedness of stimuli increases the amplitude of neural activity (e.g., early N1 and later P3 components), reflecting that self-related information recruits attention and decisional processing more strongly than other types of information [30]. However, the relationship between consciousness and self is unclear. Here, we propose that understanding how the self-prioritization effect in information processing emerges in the brain might provide a way to examine the TTC, especially the two dimensions of consciousness—temporo-spatial expansion and temporo-spatial globalization of stimulus-induced brain activity.

To better understand the neural dynamics of self-related processing, it is crucial to separate the early processes of self-saliency reflecting prepotent responses to self-related stimuli from other factors such as expectations and the familiarity of stimuli. The latter factors are often encountered in the existing literature (e.g., [31,32,33]). Therefore, it is essential to consider the distinction between the inherent automaticity of self-saliency, expectations, and stimulus familiarity [34,35], especially as these factors are twisted in various stages of information processing. To date, electroencephalogram (EEG) analyses have provided limited insights into how self-reference influences the dynamics of information processing, as most of the evidence comes from studies that used highly familiar complex stimuli (e.g., self-faces, names, knowledge, or voices) [36,37,38,39,40,41,42,43,44] and included the factor of expectation [45,46]. In these studies, it is difficult to disentangle the processes of self-saliency from the effects of stimulus familiarity and expectancies. This raises the questions of whether and how self-relatedness can mediate neural activity from the early stages of processing when expectancies and stimulus familiarity are controlled. The present study sought to elucidate this relationship, investigating how quickly self-relatedness may increase self-awareness towards self-related information and in turn modulate cognitive biases towards the stimulus.

In contrast to prior fMRI work on the self-prioritization effect, we tested a hypothesis that this effect stems from ultra-fast neural responses from early feedback modulation that enhances the saliency of self-related stimuli, progressing to later feedforward couplings for cognitive processing. The EEG approach with functional connectivity analysis was used to examine this hypothesis as it reflects a direct and real-time measurement of brain dynamics [47]. In Experiment 1, we used a shape–label matching task to measure the self-prioritization effect, controlling for differences in stimulus familiarity and complexity [44]. Participants were instructed to learn three associations between neutral geometric shapes and labels representing themself, a friend, and a stranger. After that, they immediately carried out the shape–label matching task while EEGs were recorded. Experiment 2 replicated the findings of Experiment 1, utilizing the same procedure but with the addition of a new factor of a social communicative setting (i.e., perspective taking). This new factor was used to test whether the neural dynamics underlying self-related processing were modulated by the social context.

## 2. Materials and Methods

### 2.1. Experiment 1

#### 2.1.1. Participants

Twenty participants (aged 19.9 ± 1.7 years; 8 women, 12 men) were recruited in this study. All had normal or corrected-to-normal vision. The sample size was determined from prior studies (Experiment 2B [5]). Power analysis showed that a sample of 18 participants was required to achieve a statistical power of 80% with an α of 0.001 (a two-tailed test) to detect a large effect size of the self-prioritization effect over others in the perceptual matching task (Experiment 2B [5]). Thus, a similar sample size (N = 20) was planned for the critical analysis. The experiment was approved by the local university ethics committee.

#### 2.1.2. Stimuli

Three geometric shapes (circle, square, and triangle) were randomly paired with three people (self, friend, and stranger). For example, participants were told that they would be represented by a circle, their friend a triangle, and a stranger a square. The pairing of people and shapes was counterbalanced across participants. A shape with a size of 3.8° × 3.8° was displayed above a central fixation cross of 0.8° × 0.8°, and the label (‘You’, ‘Friend’, or ‘Stranger’) of 3.1°/3.6° × 1.6° was presented below the central fixation cross. All stimuli in white were shown against a gray background. Participants had to judge whether the pairings of shape and label matched as they originally learned. The experiment was run on a PC and a 15″ CRT monitor (60 Hz refresh rate) using E-Prime in a dimly lit room.

#### 2.1.3. Procedure

Participants were invited to the laboratory so the study could be explained. The perceptual matching task was completed during the testing session over the following two weeks. The matching task consisted of two phases. The associative instruction stage took approximately 1 min, during which the participants were told the three shape–label associations, one with themselves, one with their gender-matched friend who initially joined them in the laboratory, and another with a named, gender-matched stranger. They were told, for example, that their friend would be represented by a circle, themselves by a triangle, and the stranger by a square. The stimuli were not displayed at this stage. Following the association instruction, the participants performed a shape–label matching task in which they had to judge whether the shape–label pairings matched as they were initially associated.

Each trial began with a stimulus display consisting of a shape–label pairing for 100 ms, then a jittered interval (fixation only) of 1000–1400 ms (the interval value could be realized per refresh cycle of the monitor, i.e., every ~16.7 ms). The shape–label combinations were randomly presented across trials (with an equal number of matched and mismatched trials). The participants were instructed to press the “M” and “N” keys using the index and middle fingers of the right hand as quickly and accurately as possible. Keys assigned for match and mismatch responses were counterbalanced across participants. The response time window was 1100 ms from stimulus onset. Reaction time (RT) and accuracy were recorded. After the jittered interval, feedback was provided by displaying “correct”, “incorrect”, or a “too slow” prompt for 500 ms. The trial ended with an interval (showing a central fixation cross only) of 500 ms.

The task consisted of 16 blocks, with 60 trials per block, following 12 practice trials. Thus, each participant completed 960 experimental trials (160 trials in each condition: self-matching, friend matching, stranger matching, self-mismatching, friend mismatching, stranger mismatching) with 16 short breaks. The performance for mismatching trials was calculated based on the shape (e.g., self-mismatching refers to the self-shape paired with a ‘Friend’ or ‘Stranger’ label).

#### 2.1.4. EEG Recording and Pre-Processing

EEG data were recorded using a 128-channel BioSemi ActiveTwo system (BioSemi B.V., Amsterdam, The Netherlands) at a sampling rate of 1024 Hz while participants carried out the matching task. The scalp electrodes were deployed following the 10-5 electrode nomenclature [48]. Two electrodes were deployed on the left and right mastoids for offline re-referencing. Two electrodes were placed on the outer canthi to monitor horizontal electrooculography (EOG), and another was placed below the left eye to monitor vertical EOG.

The EEG data from Experiment 1 were down-sampled to 512 Hz to reduce computational costs and remain consistent with Experiment 2. The EEG data were filtered with a 0.1 Hz high-pass filter to remove DC offsets, as well as a 50 Hz notch filter to remove the line noise. Independent component analysis (ICA) was then performed to identify and remove the artifacts. To accelerate the processing speed of ICA, CUDAICA optimization was employed to run infomax-based ICA using the graphics processing unit (GPU) [49]. After having identified and removed non-brain components, EEG data were re-referenced to the average voltage at the two mastoid electrodes. A time window of 3670 ms was then applied to segment data for a trial-based analysis, from 785 ms before the onset of the stimulus to 2885 ms after the onset of the stimulus. Data from 200 ms before to 1100 ms after the stimulus onset were chosen for further functional connectivity analysis.

**Figure 1:**
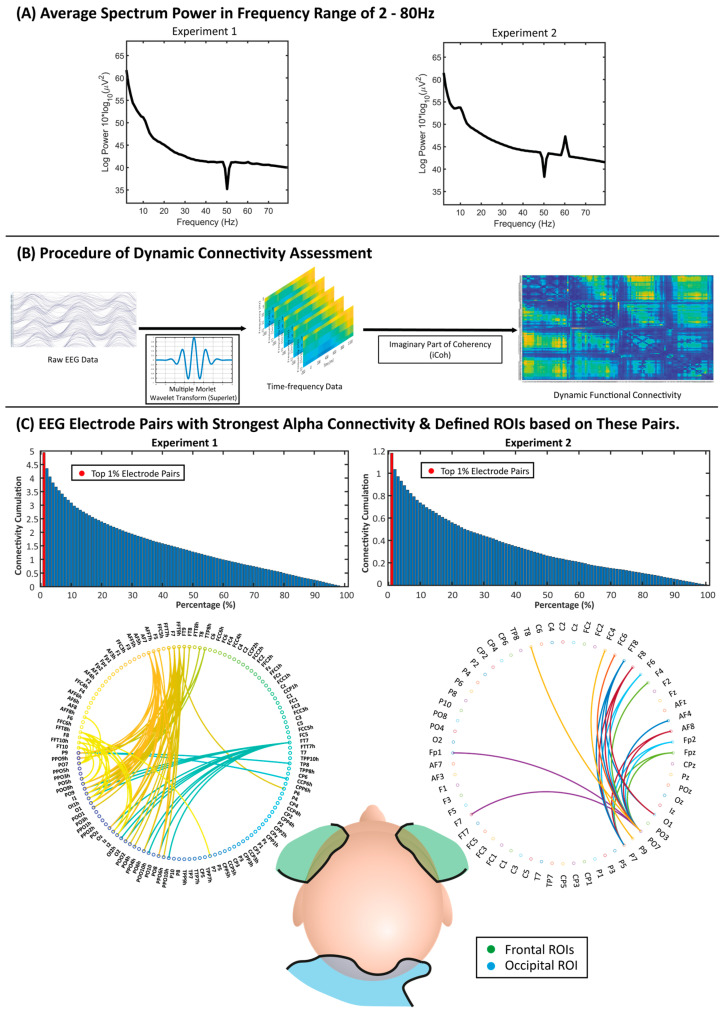
(**A**) Average spectrum power of the EEG data after pre-processing, illustrated in the frequency range of 2–80 Hz. The notches at 50 Hz were the result of the notch filters applied. In Experiment 2, the peak observed at 60 Hz was potentially due to electromagnetic noise from the CRT tube of an aged monitor (60 Hz refresh rate) used for experiment monitoring purposes by the researchers. (**B**) Procedure of assessing dynamic connectivity. EEG data were used in a time–frequency analysis with the multiple Morlet wavelet transform (Superlet). The dynamic functional connectivity between every two electrodes was then calculated by the measurement of the imaginary part of coherency (iCoh). (**C**) Histogram of absolute iCoh cumulation and the network graph of EEG electrode pairs with the top 1% average absolute iCoh in the alpha band (8–12 Hz) over the 0–1100 ms period, and the distribution of identified regions of interest (ROIs) on the head map with the distribution of selected ROIs. Colors were randomly chosen to identify different electrodes and connectivity paths.

Figure 1A illustrates the power spectrum in the frequency range of 2–80 Hz, showing the typical 1/*f* characteristics of EEG signals, in that the signal power decreases with the increase in frequency [50,51]. The alpha band (8–12 Hz) was selected for functional connectivity analysis due to the critical role of alpha oscillations in self-referential processing and visual processing [52,53,54]. Research has consistently revealed that self-referential processing, particularly when engaging the default mode network, is predominately linked to the alpha band [55]. This association is supported by the positive correlations between alpha oscillations and the default mode network, indicating a strong relationship between alpha rhythms and the neural networks that support internal self-referential processes and top-down attentional control. The current study utilized the matching task to investigate the mechanisms through which individuals link internal self-representations with external stimuli (e.g., shapes) via the modulation of attention toward socially salient stimuli. The alpha band is known for visual processing. These factors suggest that the alpha band is appropriate for investigating the neural dynamics underpinning these tasks given the tasks’ primarily visual nature. Moreover, the importance of the alpha band in neural connectivity, central to the current investigation, further validates its selection. Thus, the alpha band, with its associations with both cognitive processes and visual processing [52,53,54], provides a comprehensive framework for examining the complex neural dynamics involved in self-processing.

#### 2.1.5. Dynamic Connectivity Assessment and ROI Definition

The dynamic functional connectivity analysis for the self-prioritization effect was assessed using the EEGLAB [56] and Fieldtrip [57] toolboxes in MATLAB R2021a (Figure 1B). The Morlet wavelet, defined as a complex sine wave multiplied by a Gaussian envelope [58], was applied to pre-processed EEG data from trials of six conditions (Self, Friend, and Stranger labels with matched or mismatched responses) for the time–frequency analysis. Traditionally, a time–frequency analysis either uses a single number of signal cycles for various frequencies (ending in variable lengths of wavelet windows over different frequencies) [59] or uses a fixed wavelet window for all frequencies. However, the former approach would lead to a lower time resolution due to temporal smoothing; the latter method causes lower frequency precision towards the low frequency end due to frequency smoothing. Overall, a compromise has to be made between the time and frequency domains.

The current analysis used the algorithm “Superlet” [60] for the time–frequency analysis because it applied multiple wavelets with various numbers of cycles for each frequency, overcoming the shortcomings of traditional approaches. Specifically, wavelets with small cycle numbers could generate results with better temporal resolutions, while wavelets with large cycle numbers could generate results with higher spatial resolutions. In the current study, the width of the base wavelets was set to 3 and the length of the used wavelets in standard deviations of the implicit Gaussian kernel was set to 3. After generating a time–frequency tempo-spectrum across all EEG electrodes, the connectivity between every two electrodes was measured using the imaginary part of coherency (iCoh) [61], which is not susceptible to the issue of volume conduction in EEG data.

Due to the dense array electrode setting, further analysis was employed based on neural coupling ROIs to reduce the impact of multiple comparisons and computational burden, simplify the results, and maintain the strongest functional connectivity. The ROIs were defined on the basis of functional connectivity strengths averaged over the whole range of 0–1100 ms (the period after the onset of the stimulus), collapsed over the three matched conditions (Self matched, Friend matched, and Stranger matched) and across all participants. Among the connectivity strengths between every two electrodes, the electrode pairs with the strongest 1% iCoh values were used to identify the most powerful and representative brain regions involved in the task (0–1100 ms) (Figure 1C). Figure 1C illustrates the histogram of iCoh cumulation for electrode pairs, as well as network circles showing the distribution of top 1% electrode pairs on the brain (for the distribution of the top 5% electrode pairs on the brain, please see Figure S1 in the Supplementary Materials). Both experiments showed a similar distribution of electrode pairs showing the strongest connectivity during the experimental trials, in which they have been previously proposed to have a fundamental role in self-reference and visual processing [11,12,53,62]. Based on this, three ROIs were determined via this process for the functional connectivity analysis, two of them located in the left and right frontal regions and another located in the occipital region, namely the left-frontal, right-frontal, and occipital ROIs (Table 1). As no featured electrode pair was found between the left and right frontal regions, the neural coupling in the current experiment was between the frontal ROI (either left or right) and the occipital ROI.

The sign of the iCoh value represents the direction of dynamic functional connectivity. Specifically, positive iCoh values suggest the feedback connectivity from the frontal to occipital ROIs, while negative values suggest the opposite direction: feedforward connectivity from the occipital to frontal ROIs. Repeated-measures analyses of variance (rm-ANOVAs) were employed to statistically assess the cross-condition differences in dynamic connectivity data in every time sample. Note that the connectivity analyses focused on matching trials because the main interest of the current study was to test how quickly the brain responded to self-related stimuli, measured by matching conditions, whereas stimuli in mismatching trials were mixed up with self- and other-related information. Post hoc paired-samples *t*-tests were applied for further statistical evaluations when main effects were found in the rm-ANOVAs.

### 2.2. Experiment 2

Experiment 2 followed the same procedure as that of Experiment 1, except for including a new variable of perspective-taking and the following specifics.

#### 2.2.1. Participants

Twenty-one participants (aged 22.0 ± 4.0 years; 16 females, 5 male) with a normal or corrected-to-normal vision were invited to take part in the experiment.

#### 2.2.2. Stimuli and Procedure

The stimuli and procedure were similar to those in Experiment 1, with a few changes. The three shapes were replaced by squares with three different colors (red, green, and blue), which were associated with three labels referring to different self-relevance (You, Friend, and Stranger) [63]. The participants came to the lab once in the testing session, with no requirement to bring a friend as a companion, with the label “Friend” representing a random close friend determined by the participant before the experiment. The presentation of the colors was designed to show on the clothes of the avatars. In each trial, two avatars (4.6° × 5.2° each) facing each other were shown from a bird’s eye view along with a label (“You”, “Friend”, or “Stranger”) in the center of the screen (21″ ViewPixx LCD monitor with a 60 Hz refresh rate). One avatar was wearing a gray t-shirt and the other’s t-shirt was in one of the three associated colors (red, green, blue). Depending on the condition of a first- or third-person perspective, the avatar in the colored apparel was presented at the bottom (first-person perspective, with a bodily position aligned with the participants) or at the top (third-person perspective) of the display. Overall, the display extended a visual angle of 5.2° × 10.7°.

Twenty 60-trial blocks were employed in Experiment 2. Each trial started with a stimulus with a duration of 100 ms. Correct and incorrect associations were randomly presented with equal probabilities. The stimulus was followed by a fixation-only interval of 1200 ms. The participants were asked to judge whether the color–label association was correctly matched as they were introduced before the experiment, and then they responded by pressing two keys on the keyboard (key mapping counterbalanced across participants) within 1300 ms from the onset of the stimulus. Response feedback (“Correct”, “Incorrect”, or “Too slow”) was presented for 250 ms after the interval. The next trial started after a random inter-trial interval of 1000–1400 ms.

#### 2.2.3. EEG Analysis

EEG data were recorded using a 64-channel BioSemi ActiveTwo system (BioSemi B.V.) at a sampling rate of 512 Hz. The same procedure of EEG pre-processing as that in Experiment 1 was applied in Experiment 2 except for the down-sampling. Three ROIs were identified at similar locations in Experiment 2 compared to those identified in Experiment 1 (Figure 1C). Further statistical analysis was applied using the same rm-ANOVA methods as those used in Experiment 1 with a two-way design (Association and Perspective), as well as paired-samples *t*-tests employed between every two conditions.

## 3. Results

### 3.1. Experiment 1

#### 3.1.1. Behavioral Results

**Figure 2:**
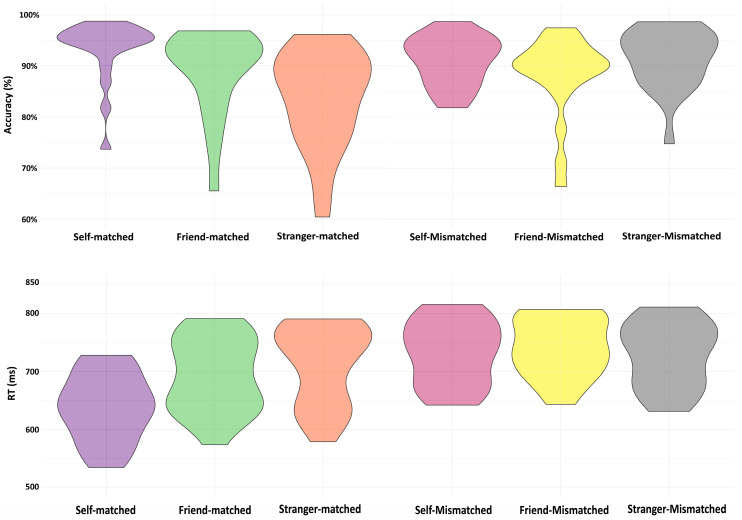
Violin plots demonstrating behavioral results for Experiment 1.

Table 2 and Figure 2 show the behavioral results, including accuracy and reaction time (RT) data in Experiment 1 (see Table 2 and Figure 2). Since the functional connectivity analyses focused on matching trials only, we report the behavioral results for matching and mismatching trials separately.

For matching trials, the ANOVAs showed significant main effects of Association on accuracy (*F*(2,38) = 23.37, *p* < 0.001). The paired-samples *t*-test analysis revealed strong evidence for higher accuracies for the self-related condition than those for friend- (*p* < 0.001) and stranger-related conditions (*p* < 0.001), and a greater accuracy for the friend-related association than the stranger-related association (*p* = 0.004). Likewise, the analysis of RTs showed a significant main effect of Association (*F*(2,38) = 40.32, *p* < 0.001), and there were faster responses to the self-related stimuli compared to those for friend- (*p* < 0.001) and stranger-related stimuli (*p* < 0.001) and shorter RTs for the friend-related stimuli than those for stranger-related stimuli (*p* = 0.04).

For mismatched trials, ANOVAs failed to show any significant effects of Association on accuracy (*F*(2,38) = 2.61, *p* = 0.09). However, there was evidence supporting a significant effect of Association on RTs (*F*(2,38) = 5.04, *p* = 0.011). Paired-samples *t*-tests showed significant differences, revealing shorter RTs for the stranger-related condition than the friend-related condition (*p* = 0.011), as well as a difference between the self- and friend-related conditions in RTs (*p* = 0.045). However, there was no evidence for any difference between the self-related and stranger-related conditions (*p* = 0.40).

#### 3.1.2. Functional Connectivity Analysis

To evaluate how early the self–other discrimination may occur, ANOVAs with the association (self, friend, stranger) for matching trials were employed to calculate dynamic connectivity between the frontal and occipital ROIs at each time sample over the range from 200 ms before to 1100 ms after stimulus onset. The analysis was conducted for the left and right frontal ROIs, respectively. To address the Type-I error inflation issue, significant effects lasting for less than ten consecutive time samples were not accepted. All statistical analyses were carried out in RStudio.

**Figure 3:**
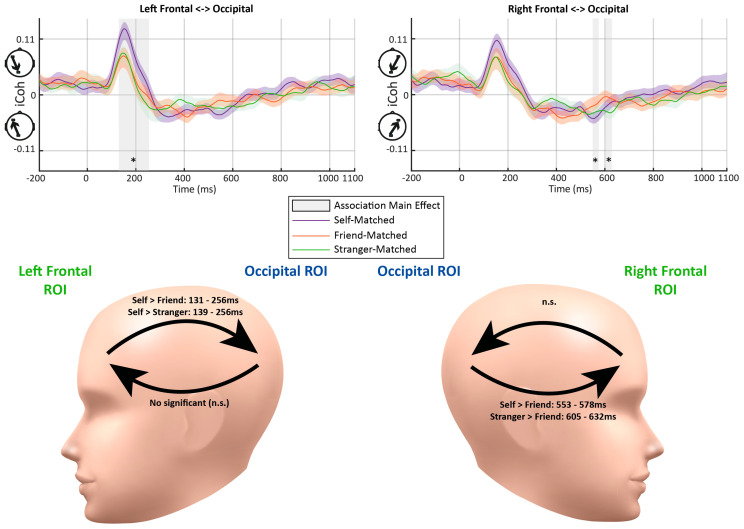
The average imaginary part of coherence (iCoh) results between the frontal and occipital regions of interest (ROIs) in matching trials in Experiment 1. The color shades represent standard errors. The vertical gray bars and asterisks indicate time periods showing significant differences (lasting more than ten consecutive time samples). The abbreviations “n.s.” represent the non-significant effect (*p* > 0.05). Significant effects of Association were detected at 131–256 ms for top-down connectivity from the left frontal to occipital ROIs. Significant effects of Association were detected at 553–578 ms and 605–632 ms regarding feedforward connectivity from the occipital to right frontal ROIs. The head models demonstrate the periods with significant differences between matching conditions in further paired-samples *t*-tests.

Figure 3 illustrates the dynamic functional connectivity (iCoh) over the time course of −200 to 1100 ms relative to the stimulus onset. Before the stimulus onset, there was a low level of connectivity from the frontal ROIs to the occipital ROI (i.e., top-down feedback connectivity). After the stimulus onset, connectivity quickly increased among all conditions until about 150 ms, when the strongest connectivity was observed in the same direction. The peak of top-down connectivity was followed by a reversion of the connectivity direction, flowing from the occipital ROIs to frontal ROIs (i.e., feedforward connectivity) from approximately 229–277 ms to about 670–820 ms. The last period showed connectivity changing back to top-down flow with a level similar to that during the baseline period. Five periods were demonstrated with significant effects across the three association conditions.

- **Early Top-down Flow**: The fronto-occipital connectivity analysis showed a rapid increase after the onset of the stimulus and achieved the peak among all conditions in the early top-down feedback period. A significant effect was detected at 131–256 ms from the left frontal to occipital ROIs (*F*s > 3.52, *p*s < 0.05) (see Figure 3). Further paired-samples *t*-tests indicated stronger connectivity for the self-related stimuli than friend-related stimuli at 131–256 ms (*p*s < 0.043) and stranger-related stimuli at 139–256 ms (*p*s < 0.048). No significant difference between the friend- and stranger-related conditions was observed (*p*s > 0.173). A similar top-down flow was also observed between the right frontal and occipital ROIs. However, no significant main effect of Association was found in this period.
- **Later Feedforward Flow**: No significant main effect of association was observed in feedforward connectivity from the occipital to left frontal ROIs (*F*s < 3.17, *p*s > 0.053). In contrast, there were significant main effects of Association on the feedforward flow from the occipital to right frontal ROIs, occurring at 553–578 ms (*F*s > 3.31, *p*s < 0.047) and 605–632 ms (*F*s > 3.26, *p*s < 0.049). The follow-up paired-samples *t*-tests showed weaker connectivity for the self-related stimuli than those for the friend-related stimuli at 553–578 ms (*p*s < 0.024). Also, a stronger connectivity for processing stranger-related stimuli was observed at 605–632 ms compared to that for friend-related stimuli (*p*s < 0.019).

#### 3.1.3. Discussion and Summary

In line with previous studies on neural mechanisms underlying self-saliency processing [64,65,66], we observed an early phase of top-down feedback connectivity, followed by a later phase of feedforward connectivity. The top-down information flow increased following the onset of the stimuli, reaching the peak around approximately 150 ms, with stronger neural couplings for self-related stimuli compared to those associated with friends and strangers, reflecting the increased self-saliency processing [5,65]. On the other hand, stimuli pertaining to strangers elicited greater connectivity during the later feedforward phases, with the connectivity for friend-related stimuli occurring earlier than that for stranger-related stimuli. This pattern of late phase connectivity might reflect cognitive processing such as selective attention and decision making [67,68,69]. Notably, a non-stimulus-induced connectivity pattern was observed prior to the onset of the stimulus. This baseline level of information flow, tracing from the frontal to occipital ROIs, indicates the presence of stable background (potentially resting-state-related) neural activities [26,27,70].

To evaluate the robustness of these dynamic neural couplings identified in Experiment 1, Experiment 2 sought to replicate these findings with a modulation of the social context. This addition was inspired by previous studies demonstrating the self-prioritization effect across various social communicative settings including personal perspective tasking [63,71].

### 3.2. Experiment 2

#### 3.2.1. Behavioral Results

**Figure 4:**
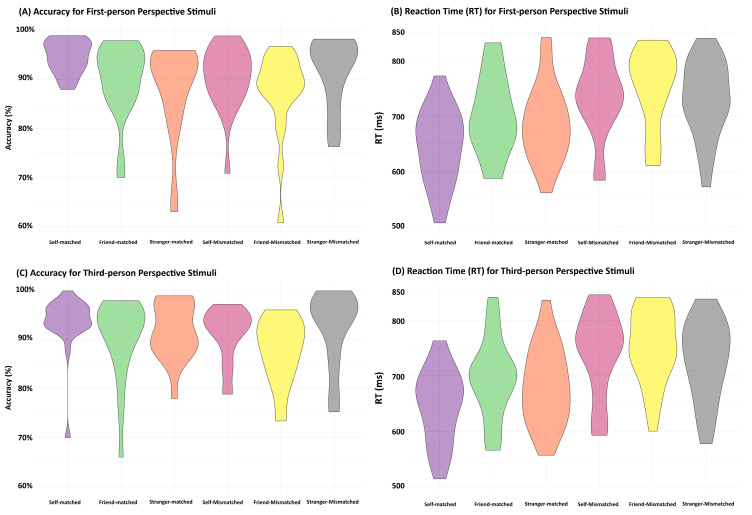
Violin plots demonstrating behavioral results for Experiment 2. (**A**,**B**) are the accuracy and reaction time (RT) for responding to stimuli in the first-person perspective. (**C**,**D**) are the accuracy and reaction time (RT) for responding to stimuli in the third-person perspective.

ANOVAs were employed to matched and mismatched results, respectively, with a two-way design, including the Association (self, friend, or stranger) and Perspective (first-person perspective vs. third-person perspective) factors. Table 3 and Figure 4 show the behavioral results.

For matching trials, the analyses of accuracy showed neither a significant interaction between Association and Perspective (*F*(2,40) = 1.25, *p* = 0.30) nor a main effect for Perspective (*F*(1,20) = 0.15, *p* = 0.70). However, there was evidence for a main effect of Association (*F*(2,40) = 5.94, *p* = 0.006), consistent with that of Experiment 1, and there was higher accuracy in processing self-related stimuli than stimuli associated with friends and strangers (*p*s < 0.002). No significant effect was observed between the friend- and stranger-related conditions (*p* = 0.15).

Neither the main effect of Perspective (*F*(1,20) = 1.40, *p* = 0.25) nor the interaction between Association and Perspective (*F*(2,40) = 0.81, *p* = 0.45) was significant in the ANOVA model using RT data. However, the model showed a significant main effect of association (*F*(2,40) = 18.04, *p* < 0.001). Further paired-samples *t*-tests revealed faster responses for self-related stimuli than stimuli associated with friends and strangers (*p*s < 0.001), and a significant difference was observed between the friend- and stranger-related conditions (*p* = 0.006).

For the mismatched data, ANOVAs on accuracy showed neither a significant interaction between Association and Perspective (*F*(2,40) = 0.16, *p* = 0.86) nor an effect of Perspective (*F*(1,20) = 0.79, *p* = 0.39). However, there was a significant main effect of Association (*F*(2,40) = 3.94, *p* = 0.03). The paired-samples *t*-tests showed greater accuracy for stranger-mismatched stimuli than those for the self- and friend-mismatched conditions (*p*s < 0.035), and no significant differences between self- and friend-mismatched conditions (*p* = 0.25) were observed.

ANOVAs of RTs failed to show a significant interaction between Association and Perspective (*F*(2,40) = 0.24, *p* = 0.79). Similarly, there was a significant main effect of Association (*F*(2,40) = 11.35, *p* < 0.001). The follow-up paired-samples *t*-tests showed significant differences between the stranger- and other-related conditions (*p*s < 0.004), but no significant difference between the self- and friend-related conditions (*p*s > 0.57). There was a significant main effect of Perspective (*F*(1,20) = 6.11, *p* = 0.02).

#### 3.2.2. Functional Connectivity Analysis

The functional connectivity pattern identified in Experiment 1 between the frontal and occipital ROIs during the presentation of stimuli was maintained in Experiment 2, irrespective of the different perspectives. In line with Experiment 1, a low level of information flow from the frontal to occipital ROIs was observed before the baseline in both the first- and third-person perspectives. After the onset of the stimulus, a similar peak in top-down connectivity enhancement was observed over all conditions. This top-down feedback connectivity was sustained up to approximately 309–359 ms, followed by a period of feedforward connectivity until around 523–625 ms. After that, the connectivity returned to a low level of top-down information flow.

**Figure 5:**
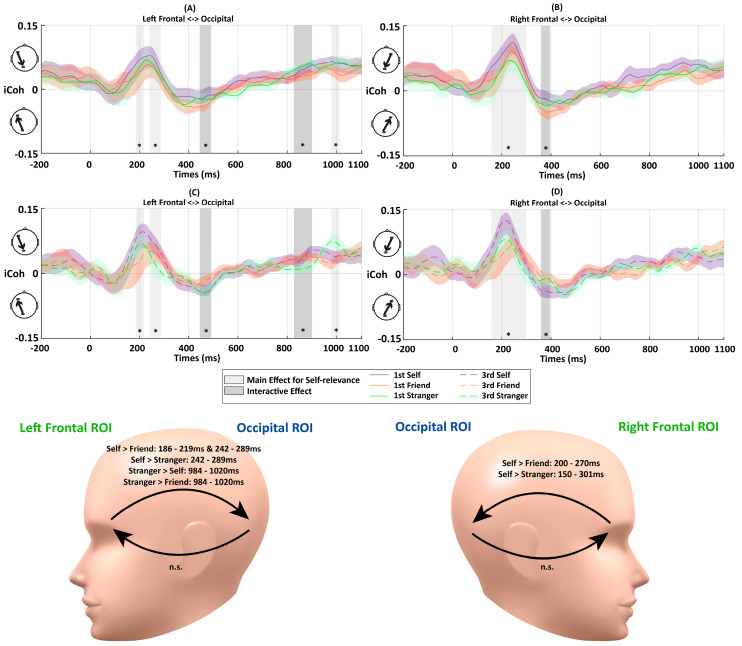
The iCoh results between the frontal and occipital ROIs during matching trials in Experiment 2. The color shades represent standard errors. The vertical gray bars and asterisks indicate time periods showing significant differences. The abbreviations “n.s.” represent the non-significant effect (*p* > 0.05). Interactions between Association and Perspective were detected at 447–494 ms and 832–906 ms between the left frontal and occipital ROIs, as well as at 361–398 ms between right frontal and occipital ROIs. Main effects of Association were detected at 186–219 ms, 242–289 ms, and 984–1020 ms between the left frontal and occipital ROIs, as well as at 150–301 ms between the right frontal and occipital ROIs. (**A**) iCoh between left frontal and occipital ROIs in the first-person perspective. (**B**) iCoh between the right frontal and occipital ROIs in the first-person perspective. (**C**) iCoh between the left frontal and occipital ROIs in the third-person perspective. (**D**) iCoh between the right frontal and occipital ROIs in the third-person perspective. The head models demonstrate the periods with significant differences between matching conditions in further paired-samples *t*-tests.

**Figure 6:**
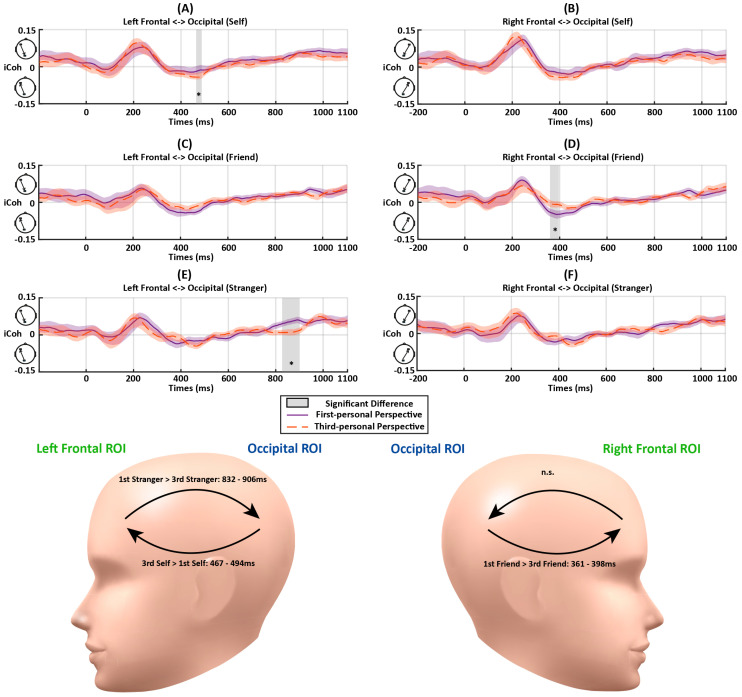
The iCoh results between the frontal and occipital ROIs during matching trials as a function of Perspective (first person vs. third person), Association (self, friend, or stranger) in Experiment 2. The color shades represent standard errors. The vertical gray bars and asterisks indicate time periods showing significant differences. The abbreviations “n.s.” represent the non-significant effect (*p* > 0.05). (**A**) iCoh between the left frontal and occipital ROIs in the self-related conditions. Increased couplings were detected at 467–494 ms in the third-person perspective (ps < 0.049). (**B**) iCoh between the right frontal and occipital ROIs in the self-related conditions. (**C**) iCoh between the left frontal and occipital ROIs in the friend-related conditions. (**D**) iCoh between the right frontal and occipital ROIs in the friend-related conditions. Decreased couplings were detected at 361–398 ms in the third-person perspective (*p*s < 0.013). (**E**) iCoh between the left frontal and occipital ROIs in the stranger-related conditions. Decreased couplings were detected at 832–906 ms in the third-person perspective (*p*s < 0.01). (**F**) iCoh between the right frontal and occipital ROIs in the stranger-related conditions. The head models demonstrate the periods with significant differences between the matching conditions in further paired-samples *t*-tests.

- **Early Top-down Flow**: There was an early top-down feedback connectivity from the frontal to occipital ROIs, consistent with Experiment 1. No significant interaction between Association and Perspective was observed for fronto-occipital connectivity (*F*s < 2.81, *p*s > 0.074). There was no significant main effect of Perspective (*F*s < 2.33, *p*s > 0.14). However, there was a significant main effect of Association at approximately 186–219 ms and 242–289 ms from the left frontal to occipital ROIs (*F*s > 3.24, *p*s < 0.049). Further paired-samples *t*-tests showed stronger neural couplings for the self-related stimuli than those for the friend- (at 186–219 ms (*p*s < 0.025); at 242–289 ms (*p*s < 0.047)) and stranger-related stimuli (at 242–289 ms; *p*s < 0.049) (Figure 5A,C).
  There was also a significant main effect of Association at 150–301 ms from the right frontal to occipital ROIs (*F*s > 3.25, *p*s < 0.049). The further paired-samples *t*-tests showed stronger connectivity for self-related conditions than those for friend- (*p*s < 0.045) and stranger-related conditions (*p*s < 0.049).
- **Later Feedforward Flow**: The early top-down feedback connectivity observed in Experiment 1 was maintained in Experiment 2 and similar direction changes of information flow were also identified. The analyses showed a significant interaction between Perspective and Association at 447–494 ms after the stimulus onset from the occipital to left frontal ROIs (*F*s > 3.43, *p*s < 0.042) (Figure 5A,C). To quantify the interaction further, paired-samples *t*-tests for the three associations were conducted for the first-person and third-person conditions, respectively. No significant difference was detected between different matching conditions in this period (*p*s > 0.05). There was stronger feedforward information flow for the self-related stimuli in the third-person perspective condition than first-person perspective condition at 467–494 ms (*p*s < 0.049) (Figure 6A).
  A similar interactive effect between Association and Perspective was observed in connectivity from occipital to right frontal ROIs at 361–398 ms (*F*s > 3.24, *p*s < 0.05) (Figure 5B,D). Further paired-samples *t*-tests showed stronger friend-related connectivity compared to those for self-related stimuli (*p*s < 0.049). In terms of the comparisons between different perspectives, a stronger connectivity for processing friend-related stimuli was observed in first-person conditions compared to those for third-person conditions (*p*s < 0.013) (Figure 6D).
- **Later Top-down Flow**: As the information flow returned to top-down feedback control at the end of the trial, there were significant interactions between Association and Perspective at 832–906 ms (*F*s > 3.26, *p*s < 0.049) from the left frontal to occipital ROIs (Figure 5A,C). There was increased top-down feedback coupling from the left frontal to occipital ROIs at 832–906 ms in the first-person perspective compared to the third-person perspective when processing stranger-related stimuli (*p*s < 0.01), but this was not the case for the self- and friend-related processing (*p*s > 0.30) (Figure 6E). Also, there was a significant main effect of Association at 984–1020 ms (*F*s > 3.30, *p*s < 0.047) from the left frontal to occipital ROIs (Figure 5B,D). The follow-up analysis showed stronger connectivity for stranger-related conditions than those for self- and friend-related conditions in this period (*p*s < 0.05). No significant difference was observed between self- and friend-related conditions in this period (*p*s > 0.11). In addition, no significant effect was observed from the right frontal to occipital ROIs in this period (*F*s < 2.43, *p*s > 0.10).

#### 3.2.3. Discussion and Summary

Experiment 2 replicated the features of the dynamic fronto-occipital neural couplings observed in Experiment 1, demonstrating the consistency of this neural connectivity. Despite the absence of an interaction between Association and Perspective in the early top-down flow, an Association x Perspective interaction was observed in the later feedforward connectivity, suggesting a modulation of neural connectivity by perspective from the occipital to left frontal ROIs. Notably, this modulation was not observed in the behavioral results.

In contrast to the findings of Experiment 1, Experiment 2 revealed an enhancement in later top-down connectivity, extending from the frontal to occipital ROIs. One possibility for these results could be due to the social communicative setting introduced in Experiment 2, which included a more complex stimulus design (i.e., avatars with different colored clothing), compared to those in Experiment 1 (i.e., basic geometric shapes). Although previous research suggests parallel mechanisms for the processing of shapes and colors in the field of visual attention [72], the complexity of stimuli in Experiment 2 may induce more complex cognitive processing.

## 4. Discussion

The present study used dynamic connectivity analyses to examine how the self-relatedness of stimuli enhances neural dynamics during information processing, thereby fostering the self-prioritization effect in cognition. The results of two experiments revealed that self-prioritization was associated with early top-down fronto-occipital connectivity and late feedforward occipito-frontal connectivity in the alpha band (8–12 Hz). Notably, the early top-down feedback connectivity was not sensitive to the stimulus complexity and social settings, highlighting its foundational role in the self-prioritization effect. Moreover, perspective taking was related to later feedforward occipito-frontal connectivity, enhancing the processing of stimuli associated with friends and strangers.

This study is the first to demonstrate the neural dynamics of self-related processing by applying the multiple wavelet transform approach (i.e., Superlet) [60] with a functional connectivity analysis utilizing the calculation of statistical dependence between EEG electrodes via the imaginary part of coherency (iCoh) [61]. This analysis revealed strong connectivity between frontal and occipital regions in the alpha band (8–12 Hz) during self-related processing, consistent with the neural framework of self-reference [44,73,74,75,76]. By controlling confounding factors—stimulus familiarity/complexity and expectancies—we showed self–other discrimination through distinct temporal processes. This discrimination was characterized by ultra-fast fronto-occipital ROI connectivity in the alpha band for self-related stimuli, compared to stimuli associated with friends and strangers. These ROIs, extensively explored in the self-processing literature [30,44,74,77,78,79,80,81], highlight the critical roles of frontal regions in self-referential processing and occipital regions in visual processing. A recent fMRI study found that neutral stimuli, such as geometric shapes associated with the self (vs. a friend), rapidly influence neural activity in the posterior intraparietal sulcus, akin to changes in perceptual salience, suggesting a modulation of social salience of stimuli [82]. In line with this finding, the current results of an enhanced early frontal-occipital connectivity for self-processing imply a top-down modulation to enhance the social saliency of self-related stimuli compared to those associated with others [20], thereby directing attention towards self-related stimuli [23].

In contrast to previous studies, the dynamic connectivity analysis pinpointed the emergence of self-awareness towards self-related stimuli in the brain. Our results demonstrated the robustness of the early top-down feedback connectivity, regardless of stimulus type and social context. Echoing the TTC account [26,27,28], this neural connectivity pattern may reflect the association of specific contents (i.e., self-related stimuli and the matching task) with consciousness initiating prior to stimulus onset and peaking approximately 150 ms after the presentation of the stimulus. Although there was no difference in associations related to oneself and others before stimulus onset, the pre-stimulus activity likely reflects the brain’s spontaneous activity/readiness for an upcoming target in the environment [66]. This continuity from pre- to post-stimulus activity may characterize shifts between different states of consciousness with varying levels of neural activity through the TTC lens [26,27,28]. In the present study, the observed enhancement in top-down connectivity for self-related stimuli may indicate a transition in states of consciousness. Specifically, it suggests a neurocognitive shift from a state of spontaneous consciousness, characterized by a baseline level of neural activity and connectivity, to a more focused and heightened state of self-awareness. This shift is triggered by the presence of self-related stimuli, evoking more elaborate and specific neural networks related to self-processing compared to stimuli linked with others. This transition implies the role of the self in organizing and directing conscious experiences. In the context of the matching task, it enhances top-down fronto-occipital connectivity, indicating a more directed and deliberate focus of attention on self-related stimuli. The temporal-spatial unfolding of this process demonstrates how certain stimuli can trigger a transition from a broad, undirected state of consciousness to a focused state of self-awareness. This dynamic interplay between temporal and spatial dimensions of brain activity provides a framework for understanding the complex nature of consciousness and its susceptibility to shifts triggered by self-relevance. Therefore, we propose that the increased top-down fronto-occipital feedback connectivity during self-related processing may serve as a marker for the emergence of self-awareness, guided by the personal significance of incoming input.

When stimuli associated with friends and strangers were presented, there was enhanced feedforward occipito-frontal connectivity observed in a later phase compared to self-related stimuli. Notably, in the matching task, the association of a neural stimulus with either a friend or a stranger enhanced the neural connectivity from occipital to frontal regions, mimicking the patterns previously delineated. This increased neural coupling may reflect processes linking visual processing to high-level cognitive functions such as attention and memory [83]. Moreover, the feedforward connectivity occurred earlier in the processing of friend-related stimuli compared to stranger-related stimuli. This result may reflect the influence of the personal relevance of stimuli on the formation of person-neutral stimulus associations [84]. This feedforward connectivity was modulated by the social communicative setting, with the first-person perspective exclusively enhancing feedforward connectivity during friend-related processing, compared to self- and stranger-related processing. In contrast, the third-person perspective specifically boosted late feedforward connectivity for stranger-related processing compared to self- and friend-related processing. This result was consistent with previous research indicating that associations with friends are particularly responsive to the activation of a first-person perspective, leading to enhanced attention towards friend-related stimuli, and subsequently, earlier feedforward connectivity [63]. On the other hand, stranger-related stimuli were often perceived from a third-person perspective, associated with a later stage of cognitive processing. Following this feedforward connectivity, Experiment 2 revealed a late feedback connectivity from the left frontal to occipital regions for stranger associations compared to self and friend associations. It is not clear what this increased top-down modulation for stranger associations means. Based on previous fMRI research, the result in Experiment 2 might be explained by increased cognitive control during more complex processing compared to Experiment 1, a possibility warranting further exploration in future studies using more difficult tasks.

Moreover, there are some implications from the current study. Notably, the features of neural couplings exhibited variations across the two experiments, influencing the robustness of the identified features. In this case, the identification of neural features was based on approximate time ranges showing featured averaging neural couplings across individuals rather than pinpointing precise timing. The observed dynamic progress in individuals with task-dependent characteristics suggests that connectivity analysis pertaining to self-processing should be investigated in a model with a larger sample size [85,86]. Moreover, the current analysis was confined to the alpha band. Indeed, previous research has shown the fundamental role of gamma activities in self-referential processing [87,88], indicating a need for further exploration into the roles of other frequency bands in the dynamic connectivity underlying self-processing. Another limitation of the current study is the lack of source analysis to pinpoint the sources of brain activities related to the self-prioritization effect [89,90,91] and a related functional connectivity analysis in the EEG source space [92,93,94,95]. This gap invites future investigations into source localization for featured regions linked to the self-prioritization effect.

There has long been interest in understanding how quickly self–other discrimination occurs in the brain. The current study indicates that such discrimination develops through ultra-fast fronto-occipital connectivity induced by the presence of self-related stimuli, irrespective of stimulus type and social context. Social context affects subsequent neural connectivity associated with cognitive processing, particularly when processing stimuli related to friends and strangers.

## Figures and Tables

**Table 1:** Electrode locations in the defined ROIs.

	Experiment 1			Experiment 2		
ROI	Left Frontal	Occipital	Right Frontal	Left Frontal	Occipital	Right Frontal
**EEG** **Electrodes**	AF7	P9	AF8	AF3	O1	AF4
	AFF5h	PPO9h	AFF6h	AF7	PO7	AF8
	AFF7h	PO7	AFF8h	FP1	P7	FP2
	F7	PO5h	F8	F3	P9	F4
	FFT7h	PO6h	FFT8h	F5		F6
	FFT9h	PO8	FFT10h	F7		F8
	FT7	PO9	FT8	FC3		FC4
	FT9	POO9h	FT10	FC5		FC6
		O1		FT7		FT8
		Oz				
		O2				

**Table 2:** Mean accuracies and RTs as a function of Association (self, friend, stranger) and Match (match vs. label-based mismatched) in Experiment 1. In the tables below, SE refers to the standard error.

Association	Match	Mean Accuracy % (SE)	Mean RTs (ms) (SE)
**Self**	**Matched**	93.3 (1.4)	638.07 (13.00)
**Friend**		88.8 (1.9)	692.95 (14.30)
**Stranger**		83.6 (2.1)	710.93 (15.33)
**Self**	**Mismatched**	89.5 (1.9)	733.01 (12.56)
**Friend**		88.2 (1.8)	742.62 (11.36)
**Stranger**		91.5 (1.3)	729.29 (12.72)

**Table 3:** Mean accuracies and RTs as a function of Association (self, friend, or stranger), Perspective (first vs. third), and Match (matched vs. mismatched) in Experiment 2.

Perspective	Association	Match	Mean Accuracy % (SE)	Mean RT (ms) (SE)
**First person**	**Self**	**Matched**	94.7 (0.7)	646.51 (14.83)
	**Friend**		90.1 (1.6)	702.84 (15.68)
	**Stranger**		88.3 (2.1)	688.17 (15.37)
	**Self**	**Mismatched**	88.8 (2.2)	766.96 (19.89)
	**Friend**		87.6 (1.8)	765.07 (17.50)
	**Stranger**		91.5 (1.6)	741.50 (16.56)
**Third person**	**Self**	**Matched**	93.5 (1.3)	645.92 (14.50)
	**Friend**		90.3 (1.8)	695.85 (16.08)
	**Stranger**		88.6 (2.5)	681.30 (15.65)
	**Self**	**Mismatched**	89.3 (2.2)	764.27 (20.18)
	**Friend**		87.7 (1.5)	759.65 (16.00)
	**Stranger**		92.3 (1.7)	733.63 (16.56)

## Data Availability

The data presented in this study are available on request from the corresponding author. The data are not publicly available due to ongoing analyses for further investigation.

## Supplementary Materials

The following supporting information can be downloaded at: https://www.mdpi.com/article/10.3390/e26030242/s1. Figure S1. Histogram of absolute iCoh cumulation (top panel) and the network graph of EEG electrode pairs (bottom panel) with the top 5% average absolute iCoh in the alpha band (8–12 Hz) over the 0–1100 ms period relative to stimulus onset. Thet top 5% iCoh values are highlighted in green in the top panel. Colors in the bottom panel were randomly chosen to identify various connectivity paths (electrode pairs).
