# Supplementary material for: Temporal Properties of Self-Prioritization
Source: Entropy (Basel). 2024 Mar 9;26(3):242. doi: 10.3390/e26030242 (PMC10969339; doi:10.3390/e26030242)
Supplement: Supplementary file 1 [file entropy-26-00242-s001.zip › entropy-2828041-supplementary.pdf]

Supplementary Document

The datasets used in the current study were collected with 128 (Experiment 1) and 64 (Experiment 2) electrode channels. Analyzing functional connectivity using all electrode pairs would be computationally impractical. Therefore, a functional connectivity analysis was performed on the basis of regions of interest (ROIs) determined by the connectivity distribution of electrode pairs showing the top 1% absolute values of the imaginary part of coherency (iCoh) in the alpha band (8–12Hz) during 1,100 ms periods from stimulus onset (0–1,100 ms relative to stimulus onsets) (see Figure 1C and Table 1). These selected activities accounted for 3.50% and 3.85% of the total connectivity activities, respectively, in Experiments 1 and 2.

To confirm the representativeness of the data selection above, we also performed an additional ROI selection by extending the iCoh value range from the top 1% to the top 5%. These selected activities accounted for 13.83% and 15.04% of the total connectivity activities in the two experiments, respectively. Despite the parameter change, a similar distribution of electrode pairs was identified (mainly fronto-occipital regions), leading to almost identical ROIs derived from the top 1% iCoh values. Figure S1 illustrates the alpha-band connectivity cumulation of electrode pairs, with the top 5% absolute iCoh values highlighted.

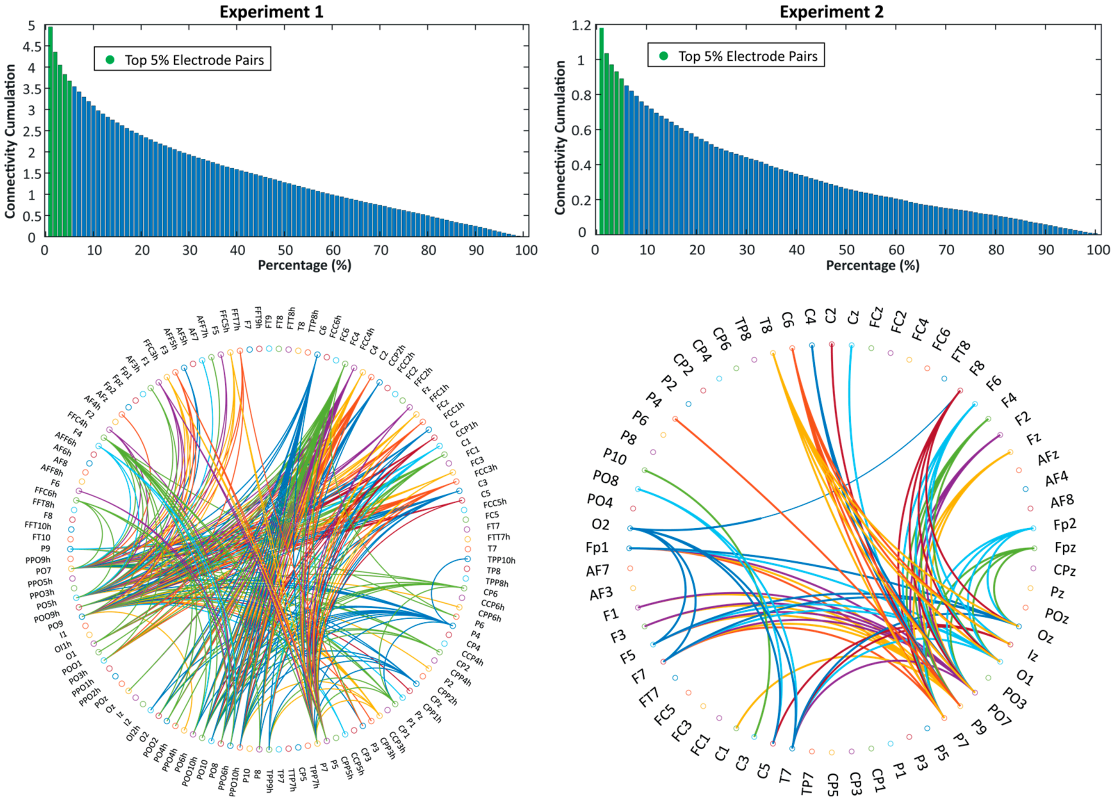

Figure S1. Histogram of absolute iCoh cumulation (top panel) and the network graph of EEG electrode pairs (bottom panel) with the top 5% average absolute iCoh in the alpha band (8–12 Hz) over the 0–1,100 ms period relative to stimulus onset. Thet top 5% iCoh values are highlighted in green in the top panel. Colors in the bottom panel were randomly chosen to identify various connectivity paths (electrode pairs).
